# AKT inhibition in the central nervous system induces signaling defects resulting in psychiatric symptomatology

**DOI:** 10.1186/s13578-022-00793-8

**Published:** 2022-05-07

**Authors:** Apostolia-Maria Tsimberidou, Antonis Skliris, Alan Valentine, Jamie Shaw, Ursula Hering, Henry Hiep Vo, Tung On Chan, Roger S. Armen, Jeffrey R. Cottrell, Jen Q. Pan, Philip N. Tsichlis

**Affiliations:** 1 Department of Investigational Cancer Therapeutics, The University of Texas MD Anderson Cancer Center, Unit 455, 1515 Holcombe Boulevard, Houston, TX 77030 USA; 2 Molecular Oncology Research Institute, Tufts Medical Center, Boston, MA 02111 USA; 3 Department of Psychiatry, The University of Texas MD Anderson Cancer Center, Houston, TX 77030 USA; 4 EMD Serono Billerica (a Business of Merck KGaA), 01821 Darmstadt, MA Germany; 5 Merck KGaA, 64293 Darmstadt, Germany; 6 Center for Translational Medicine, Thomas Jefferson University, Philadelphia, PA 19107 USA; 7 Department of Pharmaceutical Sciences, College of Pharmacy, Thomas Jefferson University, Philadelphia, PA 19107 USA; 8 Stanley Center for Psychiatric Research, Broad Institute of MIT and Harvard, Cambridge, MA 02142 USA; 9 Department of Cancer Biology and Genetics, College of Medicine, and the Ohio State University Comprehensive Cancer Center, The Ohio State University, 460 W 12th Ave, Columbus, OH 43210 USA

**Keywords:** AKT, PI3K, Advanced cancer, Clinical trial, Central nervous system, Mental illness

## Abstract

**Background:**

Changes in the expression and activity of the *AKT* oncogene play an important role in psychiatric disease. We present translational data assessing the role of AKT in psychiatric symptoms.

**Methods:**

(1) We assessed the protein activity of an *AKT3* mutant harboring a PH domain mutation (Q60H) detected in a patient with schizophrenia, the corresponding *AKT1* mutant (Q61H), and wild-type *AKT1* and *AKT3* transduced in AKT-null mouse fibroblasts and modeled the Q61H mutation onto the crystal structure of the Akt1 PH domain. (2) We analyzed the results of earlier genome-wide association studies to determine the distribution of schizophrenia-associated single-nucleotide polymorphisms (SNPs) in the *AKT3* gene. (3) We analyzed the psychiatric adverse events (AEs) of patients treated with M2698 (p70S6K/AKT1/AKT3 inhibitor) and with other PI3K/AKT/mTOR pathway inhibitors.

**Results:**

(1) Proteins encoded by *AKT3* (*AKT3Q60H*) and *AKT1* (*AKT1Q61H*) mutants had lower kinase activity than those encoded by wild-type *AKT3* and *AKT1*, respectively. Molecular modeling of the AKT1-Q61H mutant suggested conformational changes that may reduce the binding of D3-phosphorylated phosphoinositides to the PH domain. (2) We identified multiple SNPs in the *AKT3* gene that were strongly associated with schizophrenia (p < 0.5 × 10^–8^). (3) Psychiatric AEs, mostly insomnia, anxiety, and depression, were noted in 29% of patients treated with M2698. In randomized studies, their incidence was higher in PI3K/AKT/mTOR inhibitor arms compared with placebo arms. All psychiatric AEs were reversible.

**Conclusions:**

Our data elucidate the incidence and mechanisms of psychiatric AEs in patients treated with PI3K/AKT/mTOR inhibitors and emphasize the need for careful monitoring.

**Supplementary Information:**

The online version contains supplementary material available at 10.1186/s13578-022-00793-8.

## Background

The PI3K/AKT/mTOR (PAM) pathway is a highly conserved signaling cascade that plays an important role in several cellular processes. It mediates ribosome biogenesis, protein translation, autophagy, and actin dynamics. Activation of the PAM pathway by growth factors, hormones, and mitogen effectors regulates global and specific mRNA translation [1–3]. It regulates protein synthesis by directly modulating the activity of translation initiation factors and upregulating ribosome biogenesis [3, 4]. The pathway serves as a master regulator of epithelial cell metabolism, morphology, and function and integrates glycolysis with actin cytoskeletal dynamics [5]. It regulates multiple steps in glucose uptake and metabolism [6] and cytoskeletal functions including cellular movement and attachment [7]. The regulatory subunit p85α of PI3K plays a role in controlling actin dynamics associated with PDGF receptor–induced cytoskeletal changes and cell migration [8].

**Fig. 1:**
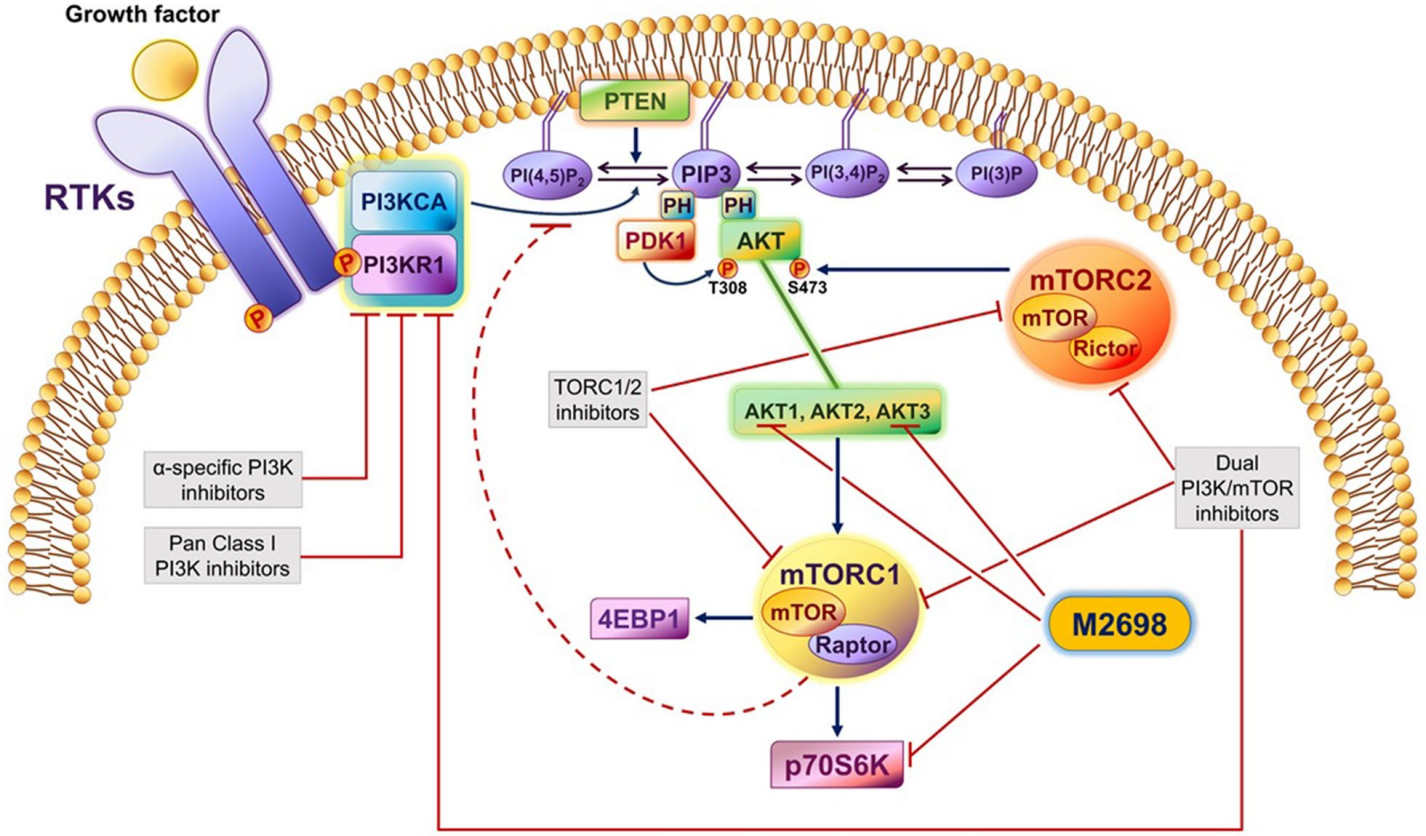
Model of the PI3K/AKT/mTOR pathway, showing the targets of pathway inhibitors. Type I PI3Ks are activated by binding via their regulatory subunits, the tyrosine phosphorylation sites of activated tyrosine kinase and other receptors in the plasma membrane. Activated PI3K catalyzes the phosphorylation of PIP2 (PI-(4,5)-P2) in the D3 position of the inositol ring to form PIP3 (PI-(3,4,5)-P3). The three AKT isoforms (AKT1, AKT2, and AKT3) translocate to the membrane by binding PIP3, where they undergo phosphorylation in the activation loop by PDK1 and in the C-terminal hydrophobic motif by mTORC2. Following this, activated AKT isoforms regulate mTORC1 and its downstream targets by multiple mechanisms. The activation of the pathway is reversed by multiple mechanisms. Prominent among these mechanisms is the removal of the D3 phosphate from the inositol ring of PIP3 by the lipid phosphatase PTEN. The targets of inhibitors of the pathway are also shown

The discovery that inhibition of glycogen synthase kinase-3 (GSK3) stabilizes mood in patients with psychiatric illness had a major impact on neuroscience [9]. Experiments addressing the regulation of GSK3 demonstrated that both known GSK3 isoforms, GSK3α and GSK3β, are inactivated by AKT via phosphorylation at Ser21 and Ser9, respectively [10], thereby confirming that inhibition of GSK3 depends on AKT activation [11, 12]. Earlier studies showed that the critical step in the catalytic activation of AKT is the binding of its PH (pleckstrin homology) domain to the D3-phosphorylated phosphoinositides PI(3,4)P2 and PI(3,4,5)P3 in the cell membrane [13]. The abundance of these phosphoinositides, in turn, depends on the activity of phosphoinositide kinases and phosphatases, primarily the type I PI3 kinase, which phosphorylates PI(4,5)P2 and PI4P in the D3 position of the inositol ring to generate PI(3,4,5)P3 and PI(3,4)P2, respectively, and the phosphatase and tensin homolog (PTEN), which removes the D3 phosphate from PI(3,4,5)P3 and PI(3,4)P2 (Fig. 1). These findings place the PAM pathway at the center of a major axis that regulates brain function and whose deregulation may lead to the development of psychiatric illness [14].

A series of observations in animal models and humans supports the hypothesis that perturbations of the PAM pathway have a role in the pathogenesis of psychiatric illness. These include schizophrenia-linked genetic polymorphisms of *ERBB4* and *NRG1* (encoding the ERBB4 ligand, neuregulin-1), deregulation of *PIK3CD* (encoding PI3Kδ) [15], activation of the dopamine 2 receptor (D2R), which inhibits AKT by activating the AKT phosphatase PP2A [16, 17], and AKT phosphorylation in postmortem studies on the brains of patients with schizophrenia or major depression [18, 19].

Genetic studies have shown that an *AKT1* haplotype associated with schizophrenia in humans resulted in low levels of *AKT1* expression and greater sensitivity to sensorimotor gating disruption by amphetamine [20]. Subsequent studies in mice showed that the ablation of *AKT1* was associated with decreased proliferation of adult-born hippocampal progenitors and with defects in hippocampal long-term potentiation, along with defects in contextual fear conditioning, spatial learning, and other hippocampus-dependent functions [18]. Ablation of *AKT2* in mice was linked to anxiety and depression-like behaviors [21], while ablation of *AKT3* resulted in small brains and schizophrenia-like symptomatology [22]. Finally, similar psychiatric illness-like defects were induced by inactivating mutations of PI3K and overexpression of PTEN, both of which result in downregulation of the activity of AKT [23, 24].

While these data provide evidence implicating dysregulation of the PI3K/AKT/mTOR axis in the pathogenesis of psychiatric illness, it is unclear whether this is the result of developmental or signaling defects. Supporting the former possibility is the evidence that PI3K/AKT/mTOR inhibition and GSK3 activation are associated with decreased neuronal cell survival [25, 26] and that, while the ablation of *AKT3* and overexpression of PTEN are associated with microcephaly (small brains) [27], high AKT3 activity and inactivating mutations of *PTEN* are associated with macrocephaly (large brains) and autism-like behaviors [23, 24]. The defects in hippocampal plasticity and function in *AKT1*^−/−^ mice [18] also suggest the possibility of a developmental defect.

A strong association between single-nucleotide polymorphisms (SNPs) in the *AKT3* gene and schizophrenia susceptibility was found by genome-wide association studies (GWAS) conducted by the Schizophrenia Working Group of the Psychiatric Genomics Consortium [28, 29]. The work presented in this report was initiated following the discovery of a missense mutation in the PH domain of the *AKT3* gene in a patient with schizophrenia in an exome sequencing study organized by the Stanley Center for Psychiatric Research at the Broad Institute (https://schema.broadinstitute.org/gene/ENSG00000117020) [30]. In that study, 97,322 healthy individuals and 24,248 patients with schizophrenia were analyzed for identification of rare genetic variants linked to schizophrenia [30]. No enrichment of coding AKT3 variants in schizophrenia patients was identified. Heterogeneity for a missense mutation in the PH domain of AKT3 (*AKT3Q60H*) was found in one patient and in none of the healthy controls. While the loss-of-function variants of AKT3 did not confer risk for schizophrenia, we characterized AKT3-Q60H because of its location in the PH domain and potential functional consequences [30].

Additionally, our earlier studies had shown that AKT3 is a strong inducer of reactive oxygen species and that its expression activates the DNA damage response and reduces cellular proliferation. As a result, cells with high expression of active AKT3 and high AKT3 kinase activity are counter-selected [31].

Therefore, we hypothesized that the AKT3-Q60H mutant may be functionally defective and have a causative role in the pathophysiology of schizophrenia. To test this hypothesis, we employed site-directed mutagenesis to introduce this mutation in the Myc-tagged *AKT3* gene, and we used retrovirus constructs of wild-type *AKT3* and the *AKT3* Q60H mutant to transduce a lung fibroblast cell line from *AKT1*^fl/fl^*AKT2*^−/−^*AKT3*^−/−^ mice we had established earlier [32, 33].

Finally, a phase I clinical trial with the brain-penetrant p70S6K/AKT1/AKT3 inhibitor M2698 (ClinicalTrials.gov: NCT01971515; EMD Serono) demonstrated that some of the M2698-treated patients developed psychiatric adverse events (AEs) and that all of these AEs were reversible [34]. Importantly, similar AEs were noted in clinical trials with other PI3K/AKT/mTOR inhibitors [35]. The reversibility of the symptoms elicited by these drugs suggests that low AKT activity in the central nervous system (CNS) may induce psychiatric illness because of signaling, rather than developmental, defects. In addition to providing information relevant to the pathophysiology of mental illness, the data in this report delineate a set of psychiatric symptoms that should be taken into consideration when designing and conducting clinical trials with inhibitors of AKT and other PAM pathway genes in human cancer.

## Results

### The AKT3-Q60H mutant, which was detected in a patient with schizophrenia, exhibits reduced catalytic activity

**Fig. 2:**
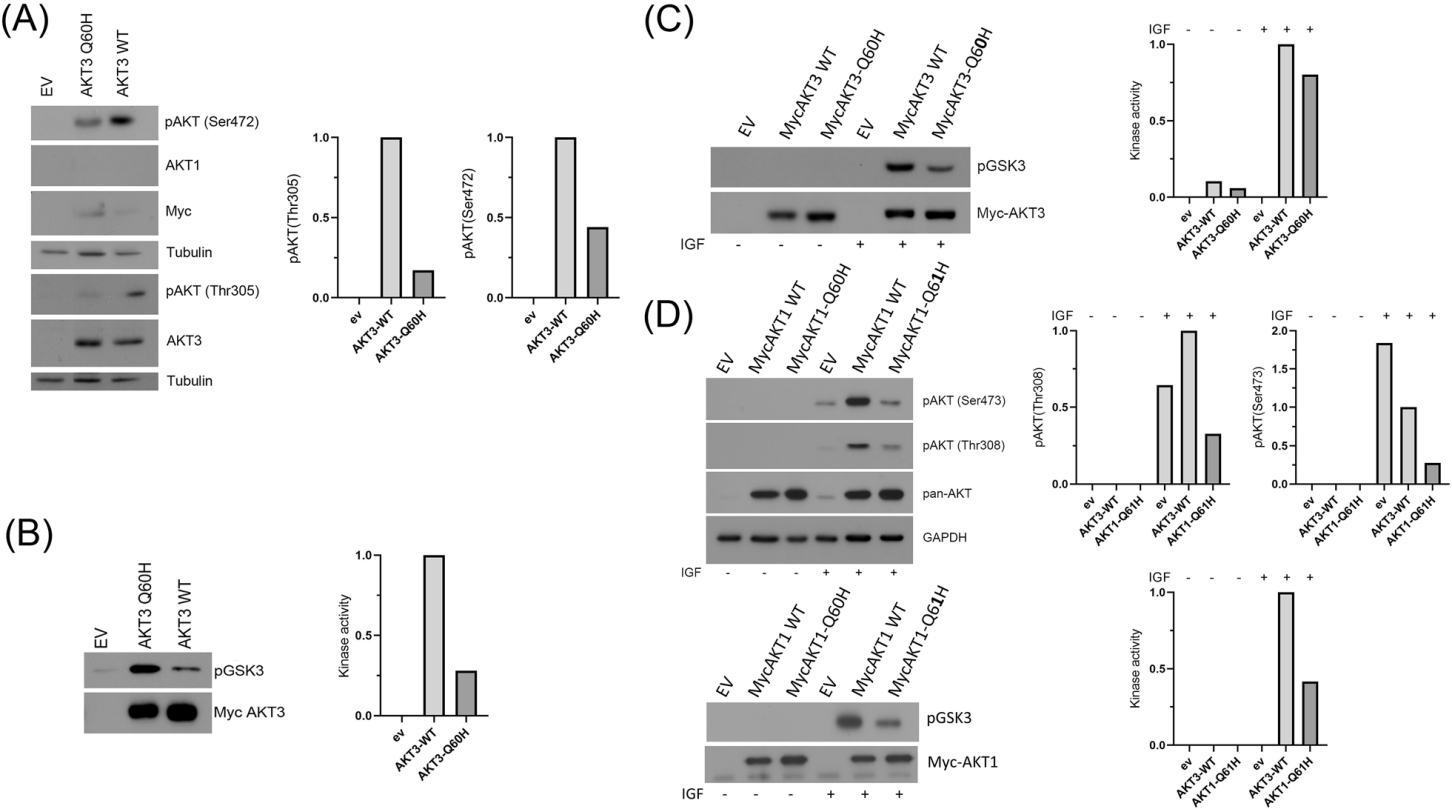
AKT3-Q60H is expressed at higher levels than wild-type AKT3 but exhibits reduced catalytic activity. **A** The indicated constructs were used to transduce spontaneously immortalized lung fibroblasts isolated from *Akt1*^*fl/fl*^*Akt2*^*−/−*^*Akt3*^*−/−*^ mice. Following this, the cells were transduced with MigR1-Cre to ablate the endogenous *Akt1*. The overall expression of AKT3-Q60H is higher than the expression of wild-type AKT3, but its phosphorylation at both Thr305 and Ser472 is reduced.** B** An in vitro kinase assay, using GSK3 as the kinase substrate, confirmed that AKT3-Q60H exhibits reduced catalytic activity. **C** The activation of AKT3-Q60H by IGF1 is impaired. Triple-AKT-knockout lung fibroblasts were transduced with the indicated constructs. Following serum starvation, the cells were treated with IGF1. In vitro kinase assays, using GSK3 as the kinase substrate, revealed that the activation of AKT3-Q60H by IGF1 is impaired. **D** *The activation of AKT1-Q61H by IGF1 is also impaired.* Upper panel. Triple-*Akt*-knockout lung fibroblasts were transduced with the indicated constructs. Following serum starvation, the cells were treated with IGF1. Probing Western blots of the cell lysates with antibodies recognizing the phosphorylated activation loop (Thr308) or the phosphorylated C-terminal hydrophobic motif revealed that the activation of AKT1-Q61H by IGF1 is also impaired. Lower panel. In vitro kinase assays, using GSK3 as the kinase substrate, confirmed the low enzymatic activity of AKT1-Q61H in IGF1-treated cells. The retroviral vector for the constructs used in these experiments was pBabe-puro. Quantification of the Western blot bands was performed using ImageJ software. For the kinase assays (panels B, C, and lower part of D), we calculated the ratio of phosphorGSK3 to the corresponding wild-type Myc-AKT3 or Myc-AKT1, and we gave these ratios the value of 1. The phosphor/total ratios of the mutant forms were calculated relative to the ratios of the wild-type forms. For wild-type AKT3 and AKT1 phosphorylated at Ser472/Ser473 or Thr305/Thr308, the ratios of phosphorAKT3/myc-AKT3 and phosphorAKT3/AKT3 (panel A) or phosphorAKT1/pan-AKT (upper part of panel D) were calculated and given the value of 1. The ratios of the mutant forms of AKT3 and AKT1 were again calculated relative to the ratios of the wild-type forms. The experiments in Figs. 2A and 2D were performed twice. The experiment in Fig. 2C was in part a repeat of the experiment in 2B. Importantly, the kinase experiments also confirmed the results of the Western blotting experiments, and the high expression of the AKT3-Q60H mutant provided additional strong evidence for the impaired enzymatic activity of the mutant

We found that the phosphorylation of AKT3-Q60H at both the Thr305 and Ser472 sites was impaired (Fig. 2A), suggesting that the mutant protein exhibits reduced catalytic activity. In addition, the expression of the mutant protein was higher than that of the wild-type protein. In vitro kinase assays, using GSK3 as the kinase substrate, demonstrated that the kinase activity of AKT3-Q60H was impaired (Fig. 2B). In vitro kinase assays using AKT3 immunoprecipitated from cell lysates harvested 15 min later and GSK3 as the kinase substrate demonstrated that the activation of the AKT3-Q60H mutant by IGF1 was also impaired (Fig. 2C). Interestingly, the corresponding AKT1 mutant (Q61H) also exhibited reduced catalytic activity, as evidenced by the low level of phosphorylation of the protein at Thr308 and Ser473 (Fig. 2D).

### Modeling of the binding of PI(3,4,5)P3 to the wild-type and mutant AKT PH domain

**Fig. 3:**
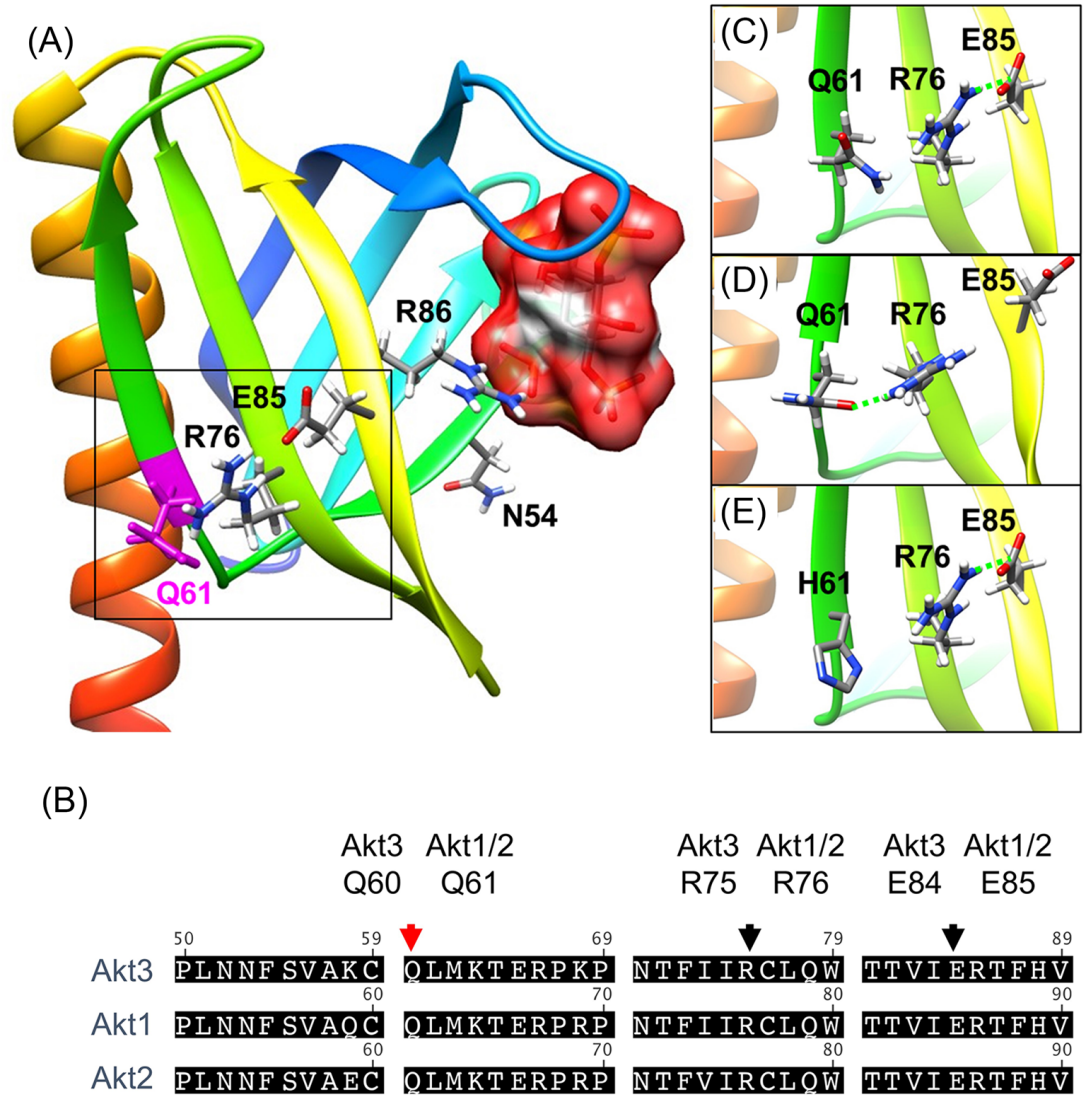
Modeling the Q61H mutation onto the crystal structure of the Akt1 PH domain. **A** The structure of the Akt1 PH domain in complex with Ins(1,3,4,5)-tetrakisphosphate [1h10.pdb]. The ribbon structure is rainbow colored, from blue at the N-terminus to red on the C-terminal helix (res: 93–114). The bound Ins(1,3,4,5)-tetrakisphosphate is shown with a molecular surface. Key side chain residues Q61, R76, and D85 are displayed as sticks, where Q61 is highlighted in magenta. **B** Amino acid homology comparison between human Akt1, Akt2, and Akt3 kinases. Key side chain residues of Akt3 Q60, R75, E84 (Akt1 and Akt2 Q61, R76, E85) are indicated. **C** Zoom-in view of the crystallographic conformation of Q61, R76, and E85, where a strong salt bridge electrostatic interaction is formed between R76 and E85. **D** An alternative conformation of the Q61 side chain from 100 ps molecular dynamics simulation of the PH domain. This alternative conformation illustrates how the carbonyl group of the Q61 side chain is able to form favorable side chain interactions with R76. The hydrogen bonding interaction shown with a green dashed line is characterized by a 2.74 Å distance (between Q61@OE1 and R76@NH1). **E.** Modeled low-energy conformation of the Q61H mutation into the crystallographic conformation

We found that the Q61 residue is located in the β5-strand of the PH domain, and its two major side-chain interactions are hydrophobic interactions with the (i,- > i + 2) residue M63 (in the β5 strand) and the R76 residue in the adjacent β6 strand (Fig. 3A). Importantly, the Q61 and R76 residues, as well as the E85 residue in the β7 strand, are conserved among all three AKT isoforms (Fig. 3B). In the crystallographic conformation, there were no favorable polar interactions between the Q61 and R76 side chain, but there was a very strong electrostatic salt bridge between R76 and E85 (Fig. 3C). However, two complementary modeling approaches, molecular dynamics simulations and analysis of a side-chain rotamer library, both show that the carbonyl group of the Q61 side chain may form favorable polar interactions with the guanidino side chain of R76 (Fig. 3D), while the Q61H histidine side chain rotamers are not able to form equivalent favorable interactions (see Additional file 1: Methods). In addition, partially charged protamers of the H61 imidazole side chain, expected over a pH range of 6.0 to 7.0, will result in repulsive charge-charge interactions with R76 (see Fig. 3E) that may significantly affect the structure and dynamics of the PH domain. Finally, observed structural changes to the conformational state of the Q61H binding pocket residues (K14 and R86) in molecular dynamics simulations support the hypothesis that Q61H may reduce the binding affinities for D3 phosphorylated phosphoinositides.

### Common SNPs link AKT3 to the development of mental illness

**Fig. 4:**
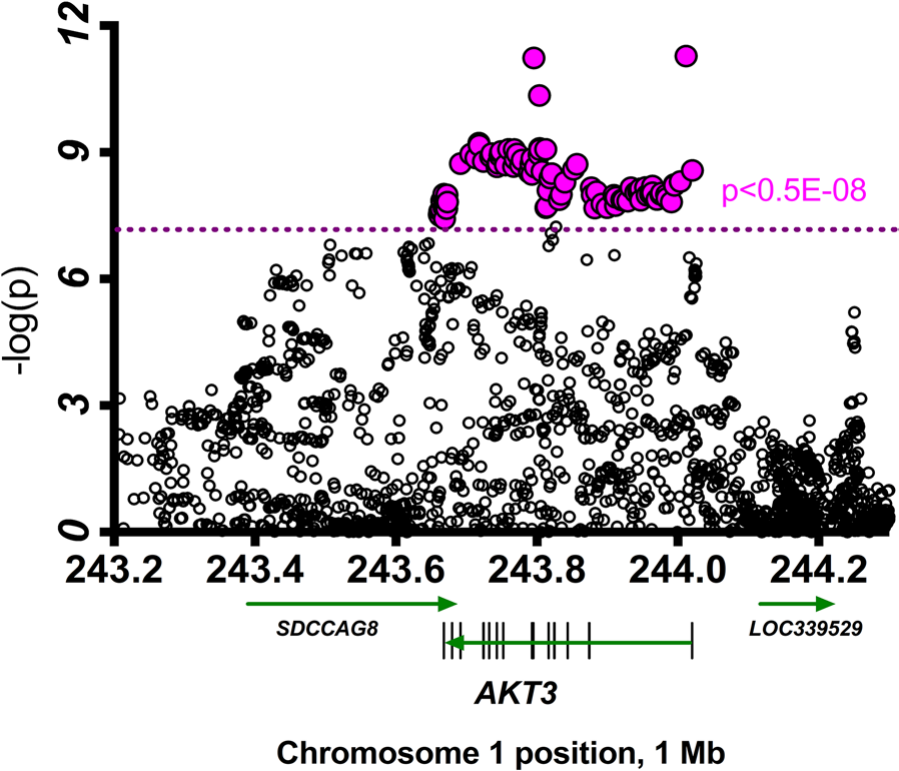
Common SNPs across the AKT3 gene significantly associated with schizophrenia. Pink dots: SNPs with genome-wide significant association with schizophrenia risk (p < 5 × 10^–8^). The peak risk association SNP is rs61833239 in this region (p = 5.22 × 10^–12^). Of note, there were no variants in the coding region of AKT3 that were significantly associated with schizophrenia risk. The SNPs that were significantly associated with risk of schizophrenia were all in the non-coding region of AKT3

Analyzing the publicly available dataset of the schizophrenia study [28, 29], we identified multiple SNPs with a genome-wide significant association with schizophrenia (p < 0.5 × 10^–8^). All schizophrenia-linked SNPs in the chromosomal region centered around *AKT3* map within the *AKT3* gene itself (Fig. 4). The peak risk association SNP in this region was rs61833239 (p = 5.22 × 10^–12^).

### The use of PI3K/AKT/mTOR pathway inhibitors in patients with cancer gives rise to reversible psychiatric symptomatology

Table 1 illustrates the psychiatric adverse events noted in patients with advanced cancer treated on a phase I study of the brain-penetrant p70S6K/AKT1/AKT3 inhibitor M2698. Overall, 29% (29/101) of treated patients developed psychiatric AEs: M2698 monotherapy, 26% (16/62); M2698 plus tamoxifen, 35% (9/26); and M2698 plus trastuzumab, 31% (4/13). The most frequent adverse events were insomnia, anxiety, and depression. All psychiatric AEs were reversible and manageable with dose reduction. Some patients developed multiple psychiatric AEs. Five patients contributed to 40% of all AEs in the study. Of the 29 patients who developed psychiatric AEs during the study, 45% (13/29) had no prior psychiatric symptomatology. Details of psychiatric AEs in selected patients are described in Additional file 1: Results. Additional file 1: Table S2 describes patients who reported multiple (≥ 3) psychiatric AEs with M2698 treatment.

**Table 1:** Psychiatric adverse events in patients with advanced cancer treated on a phase I study of the brain-penetrant p70S6K/AKT1/AKT3 inhibitor M2698

Dose of M2698 (mg/day)	Patients in cohort (n)	Patients with AEs (n)	Psychiatric AEs in cohort (count)	Number of treatment-emergent adverse events (any grade)							
				Abnormal dreams/nightmares/insomnia/sleep disorders	Anxiety/emotional distress	Depression/pathy/ euphoric mood/ mania	Delusion/ paranoia	Hallucination/auditory hallucination	Reading disorder	Confusional state	Affect lability
*Mono-therapy*											
15	3	0	0	0	0	0	0	0	0	0	0
30	3	0	0	0	0	0	0	0	0	0	0
60	6	2	2	0	1	0	0	0	0	0	1
75	4	1	2	2	0	0	0	0	0	0	0
110	4	0	0	0	0	0	0	0	0	0	0
160	6	2	3	2	0	1	0	0	0	0	0
200	3	1	1	0	1	0	0	0	0	0	0
240	17	5	5	2	1	2	0	0	0	0	0
320	12	4	9	4	2	1	2	0	0	0	0
380	4	1	1	0	1	0	0	0	0	0	0
*M2698 (*+ *tamoxifen)*											
80	4	4	9	1	4	0	1	1	1	1	0
160	9	2	3	1	1	1	0	0	0	0	0
200	6	2	3	2	1	0	0	0	0	0	0
240	7	1	2	1	0	1	0	0	0	0	0
*M2698 (*+ *trastuzumab)* *0* *0*											
80	4	1	1	0	0	1	0	0	0	0	0
160	9	3	12	3	3	4	1	1	0	0	0
**TOTAL**	**101**	**29**	**53**	**18**	**15**	**11**	**4**	**2**	**1**	**1**	**1**

Overall, 29% (29/101) of the patients reported 53 psychiatric treatment-emergent AEs without evident M2698 dose dependency. Some patients developed multiple psychiatric AEs. Notably, 5 patients, who had 3 to 5 psychiatric AEs each, accounted for 40% (21/53) of psychiatric AEs (Additional file 1: Table S2). The most affected cohort was the trastuzumab cohort, where 3 patients reported 12 AEs at the highest dose level (160 mg). In the M2698 monotherapy cohort at a dose level of 320 mg and in the trastuzumab combination cohort at a dose level of 80 mg M2698, one patient each had 5 and 4 AEs, respectively. A medical history of psychiatric symptoms was documented in 56/101 (55%) patients corresponding to the observed AEs in the trial, i.e., mainly anxiety, depression, and insomnia

As of August 2020, more than 825 clinical trials with PAM pathway inhibitors, targeting single or multiple enzymes, have been conducted in patients with cancer. Table 2 summarizes the psychiatric AEs reported in selected randomized clinical trials, comparing treatment arms with or without PI3K/AKT/mTOR inhibitors. Although psychiatric AEs were not reported in clinical trials of alpelisib, capivasertib (AZD5363), or ipatasertib, they were noted in trials of buparlisib, taselisib, and MK-2206 in patients with breast and prostate cancer. Among these last three drugs, the highest incidence of mood disorders, including depression, mania, anxiety, and suicide attempts, was observed during treatment with buparlisib, which crosses the blood–brain barrier. In addition to the psychiatric AEs, whose incidence was higher in the PAM inhibitor arms than in the placebo arms, there was also an increased incidence of neurological symptoms such as dizziness and headache in the treatment groups across all studies. Headache was more frequent in taselisib- and MK-2206-treated patients.

**Table 2:** Psychiatric adverse events reported in selected randomized clinical trials comparing treatment arms with or without PI3K/AKT/mTOR inhibitors

Adverse Event	*	NCT01633060		NCT01572727		NCT02340221		NCT01251861	
		Placebo + Fulvestrant	Buparlisib + Fulvestrant	Placebo + Paclitaxel	Buparlisib + Paclitaxel	Placebo + Fulvestrant	Taselisib + Fulvestrant	Obs. + Bicalutamide	MK-2206 + Bicalutamide
		n = 140	n = 288	n = 201	n = 202	n = 213	n = 416	n = 50	n = 53
**Psychiatric symptomatology**									
*Impaired reality testing*									
Acute psychosis	S	N.R	N.R	0.00%	0.50%	N.R	N.R	N.R	N.R
Psychotic disorder	S	N.R	N.R	0.00%	0.50%	N.R	N.R	N.R	N.R
*Delirium*									
Confusional state	S	0.71%	1.39%	0.50%	0.99%	0.00%	0.24%	N.R	N.R
Delirium	S	N.R	N.R	0.00%	0.50%	N.R	N.R	N.R	N.R
Disorientation	S	0.00%	0.69%	N.R	N.R	N.R	N.R	N.R	N.R
Mental status changes	S	N.R	N.R	0.00%	0.99%	0.00%	0.48%	N.R	N.R
*Mood disorders*									
Depression	S	N.R	N.R	0.00%	0.50%	N.R	N.R	N.R	N.R
	NS	7.86%	20.83%	8.46%	24.75%	N.R	N.R	N.R	N.R
Mania	S	0.00%	0.35%	N.R	N.R	N.R	N.R	N.R	N.R
Mood altered	S	0.00%	0.35%	N.R	N.R	N.R	N.R	N.R	N.R
	NS	1.43%	5.21%	2.49%	5.45%	N.R	N.R	N.R	N.R
*Other*									
Anxiety	S	N.R	N.R	0.00%	0.50%	N.R	N.R	N.R	N.R
	NS	10.71%	17.71%	15.42%	20.30%	N.R	N.R	N.R	N.R
Mental disorder	S	0.00%	0.35%	0.00%	0.99%	N.R	N.R	N.R	N.R
Suicide attempt	S	0.00%	1.04%	N.R	N.R	N.R	N.R	N.R	N.R
**Neurologic symptomatology**									
Dizziness	S	0.00%	0.35%	N.R	N.R	0.00%	0.72%	N.R	N.R
	NS	7.14%	12.15%	10.45%	16.34%	8.45%	9.86%	2.00%	7.55%
Migraine	S	0.00%	0.35%	N.R	N.R	N.R	N.R	N.R	N.R
Headache	NS	10.71%	9.38%	18.41%	17.33%	11.74%	19.95%	6.00%	11.32%
Resting tremor	S	0.00%	0.35%	N.R	N.R	N.R	N.R	N.R	N.R
Tremor	NS	0.00%	6.25%	N.R	N.R	N.R	N.R	N.R	N.R

^*^Serious (S): Results in death, is life-threatening, requires inpatient hospitalization or prolongs hospital stay, causes significant incapacity, interferes substantially with daily activities, or causes a congenital anomaly or birth defect. Non-Serious (NS): An adverse event that is not a serious adverse event. N.R., not reported. Obs., observation

## Discussion

The detection of a functionally hypoactive *AKT3* mutation in a patient with schizophrenia, combined with the GWAS results linking *AKT3* SNPs with schizophrenia, suggested a link between the *AKT3* gene and schizophrenia. A third piece of evidence confirming this association was provided by the AE data from a phase I study of the brain-penetrant p70S6K/AKT1/AKT3 inhibitor M2698 (EMD Serono) [34] and by a review of psychiatric AEs reported in clinical trials of other PAM pathway inhibitors.

Our results suggest that decreased AKT activity in the CNS, caused by mutations (such as *AKT3Q60H*), by genetic polymorphisms in the AKT3 gene, or by treatment with brain-penetrant PAM pathway inhibitors, promotes the development of psychiatric symptoms in some individuals. The activity of the mutant AKT protein was reduced and its expression was higher than that of the wild-type protein in the cellular construct. In addition, psychiatric symptoms induced by pharmacological blockade of the pathway were reversible, suggesting that its emergence may depend on signaling, rather than developmental, defects. These data contribute to the understanding of the role of the AKT pathway in the pathophysiology of psychiatric illness. Furthermore, the association of psychiatric symptomatology with PAM pathway inhibitors suggests that efficient development of this important class of anticancer drugs requires standardized methods to assess, monitor, and mitigate the risk of these events in clinical trials.

Although the activity of AKT in the CNS was not monitored during the M2698 clinical trial, experiments in mouse models have shown that M2698 crosses the blood–brain barrier, resulting in CNS exposures sufficient to inhibit AKT1, AKT3, and p70S6K in vitro [36]. Moreover, in the phase I study, M2698 was shown to strongly inhibit AKT1, AKT3, and p70S6K in the tumor tissue. Finally, M2698 was found to exhibit robust pharmacodynamic activity toward p70S6K and AKT in post-treatment brain tumor biopsies of orthotopic xenograft mouse models. These observations collectively suggest that M2698 inhibits AKT in the brain, although we understand that the tumor data may not be directly translatable to the normal brain as these tissues may differ in vascularity, which would affect the concentration of the drug, and in the genetic and epigenetic regulation of the target pathway, which may affect the response to the drug.

The psychiatric AEs observed with M2698 treatment are consistent with observations in clinical trials of other PAM pathway inhibitors [35]. For instance, a phase Ib/II study of pictilisib (GDC-0941), a pan-PI3K inhibitor, combined with cisplatin in androgen receptor-negative, triple-negative metastatic breast cancer was closed early owing to gastrointestinal and neuropsychiatric toxicities, and the phase III studies of buparlisib, a pan-PI3K inhibitor, in hormone receptor-positive breast cancer demonstrated a high incidence of treatment-related psychiatric symptomatology, which halted further development of this molecule [37, 38]. An important factor that may be responsible for differences in the incidence and severity of symptoms induced by different inhibitors is the differential ability of these inhibitors to cross the blood–brain barrier. The host factors responsible for the heterogeneity in the penetrance and presentation of these AEs are unknown. To identify these factors, we need to discover biomarkers associated with predisposition to these events, which could include germline polymorphisms in *AKT* (primarily *AKT3*) and in other pathway-specific regulatory molecules. Such studies will benefit from the development of methodologies to monitor AKT activity in the CNS.

There are several challenges in interpreting psychiatric AEs in the context of oncology clinical trials and in comparing or combining data from different trials. First, it is difficult to attribute low-prevalence events to a study drug when there is a high background of these events in the patient population at baseline. Second, psychiatric AEs are not consistently reported across trials, and therefore standardization of reporting is needed for consistent monitoring of these events in order to mitigate drug toxicity and support the development of this class of therapeutics. Because behavioral AEs occur in patients who are already vulnerable to such symptoms owing to other organic and functional stressors, it will be beneficial to include experienced psycho-oncology specialists in the treatment team. Neuropsychological testing may also be useful to assess subclinical vulnerability before treatment, as well as the extent of the recovery from or the chronicity of deficits afterwards. Currently, our clinical data suggest that it is possible for patients with a history of psychiatric disorders to successfully participate in such clinical trials if they are properly monitored and managed, thereby expanding the benefit of these targeted therapies to a broader population of patients.

Our observations are in line with published data demonstrating an association between the PAM pathway and psychiatric illness [39]. PAM pathway disruption of protein synthesis and actin dynamics can lead to abnormal neuronal morphology, learning/memory deficits, and psychiatric disease [19, 22, 40–45]. Proteins implicated in this pathway are downregulated in the dorsolateral prefrontal cortex in schizophrenia [40]. In murine models, decreased AKT activity in the ventral tegmental area was associated with increased susceptibility to depressive behavior [45]; antipsychotic drugs were shown to activate GSK3β-AKT signaling [41–43]; and GSK3β inhibitors had anti-depressant effects in animal models [44]. Furthermore, AKT isoform- and gender-specific effects were seen on levels of anxiety, spatial and contextual memory, and fear extinction [46]. Selected AKT SNPs have been associated with psychiatric disease [20, 46–49]. For instance, the SNPs rs1130214 and rs3730358 have been associated with higher Akt1 level, bipolar disorder, and major depression [20, 50, 51]. In patients with depressive disorders, the polymorphism rs1130214 was associated with response to antidepressant therapy [52].

AKT3 may function as a key regulator of PI3K/AKT/mTOR signaling in the brain. Although there is no direct evidence that AKT3 protein levels are reduced in patients with schizophrenia, one study showed that the relative phosphorylation of GSK3β at Ser9 (downstream of all AKTs) was significantly lower in lymphocytes and in the frontal cortexes of individuals with schizophrenia compared with controls [20], providing indirect evidence that AKT3 could be reduced in their brains as well. AKT3 deletion associated with decreased GSK3α/β phosphorylation levels in various brain regions was restored with chronic administration of lithium, a mood stabilizer. Lithium treatment also rescued depressive and anxiety-like behaviors in AKT3-knockout mice [53]. AKT3 may be the primary target for mTORC2 activity, as ablation of the mTOR2 complex proteins Rictor and Sin1 was shown in mice to decrease AKT Ser^473^ activation, causing microcephaly and alterations in neuronal morphology and function [22].

To date, only six PAM pathway inhibitors have been approved by the US Food and Drug Administration (FDA): the mTOR inhibitors everolimus and temsirolimus, the PI3K-δ inhibitors idelalisib and duvelisib, the PI3K-α inhibitor alpelisib, and the pan-PI3K inhibitor copanlisib (Additional file 1: Table S3). The main challenge for the development of these therapeutics is toxicity, which includes skin rash, hyperglycemia, and gastrointestinal and psychiatric symptoms [54]. These adverse events require treatment interruption, with or without dose reduction, and in severe cases, treatment discontinuation.

Given the complexity of the PAM pathway and clinical translation of our data, our study has several limitations. First, lung fibroblasts were utilized to study the impact of AKT mutations on kinase activity, whereas a more relevant model would be primary neurons. However, AKT activation via PIP3 binding to the PH domain occurs in all cells, including fibroblast and neuronal cells. A structural change in the PH domain will, therefore, affect a function that is common in all cell types. Consequently, the activity of this AKT3 mutant is expected to be similar in fibroblasts and neuronal cells. Second, we cannot determine whether patients treated with M2698 who experienced psychiatric AEs [34] also carried SNPs in AKT3, making them theoretically more susceptible to the study drug. SNP analyses should be planned prospectively in future clinical trials with PAM pathway inhibitors. Third, although our data suggest that the elicitation of reversible psychiatric symptoms by acute PI3K/AKT/mTOR inhibition may be due to signaling defects, this observation has to be interpreted with caution. Since AKT is a central node in a multitude of important cellular pathways, perturbation of brain development caused by a genetic mutation that impairs AKT kinase activity cannot be excluded.

## Conclusions

In summary, we provide genetic data and data derived from clinical trials that further elucidate the role of AKT inhibition in psychiatric symptomatology. Our structure–function studies addressing the effects of an AKT PH domain mutation observed in a patient with schizophrenia deepen our understanding of AKT regulation by D3 phosphorylated phosphoinositides. Future research should focus on the identification of covariates that contribute to the development of psychiatric symptomatology in individuals treated with PAM pathway inhibitors and on the determination of the frequency of AKT activity downregulation among patients with psychiatric symptoms. To address these questions, methods to assess the risk and monitor the development of psychiatric symptomatology should be developed and standardized across clinical trials of PAM pathway inhibitors, and non-invasive technologies should be developed to monitor the activity of AKT in the CNS.

## Methods

### Cells and constructs

The methodology used was as previously published [32]. The immortalized *AKT1*^fl/fl^*AKT2*^−/−^*AKT3*^−/−^ lung fibroblasts were transduced with pBabe-puro/Myc-Akt3, pBabe-puro/Myc-Akt3-Q60H, pBabe-puro/Myc-Akt1, or pBabe-puro/Myc-Akt1-Q61H constructs or with the pBabe-puro empty retroviral vector. The constructs used in the experiments in this report have been described previously [32]. Cells transduced with the empty vector were used as a control. Subsequently, all cells were superinfected with a MigR1-Cre construct to ablate the floxed endogenous *AKT1* gene [32]. The triple-knockout cells that were not rescued with *AKT3* did not proliferate, but they did remain alive for 7 to 10 days, so they could be used to determine response to external signals. Details about the experiments and sources of cells, constructs, etc. are provided in Additional file 1: Methods.

### Site-directed mutagenesis

Site-directed mutagenesis was performed using polymerase chain reaction–based procedures, and pairs of overlapping oligonucleotide primers harboring the AKT3-Q60H or the AKT1-Q61H mutation were used to amplify pBabe-puro constructs of AKT3 and AKT1, respectively.

### Immunoblotting

Cell lysis and protein extraction were performed using standard procedures. Western blots of lysates from cells maintained in complete serum-containing media were probed with antibodies to AKT1 and AKT3, the Myc-tag antibody, and antibodies that recognize the phosphorylated Thr305 and Ser472 sites of AKT3 (corresponding to the AKT1 phosphorylation sites Thr308 and Ser473). The antibodies used are listed in Additional file 1: Table S1.

### In vitro kinase assay

To determine whether the AKT3-Q60H mutant responds to tyrosine kinase receptor-initiated signals, the triple-*AKT*-knockout lung fibroblasts rescued with wild-type or mutant AKT3 were serum-starved and stimulated with IGF1. Immortalized *AKT1*^fl/fl^*AKT2*^−/−^*AKT3*^−/−^ lung fibroblasts transduced with pBabe-puro/myc-Akt3 (wild-type or Q60H-mutant) or pBabe-puro/myc-Akt1 (wild-type or Q61H-mutant) and with MigR1-Cre were lysed with the Triton X-100 buffer. In vitro kinase assays on Myc-Akt3 immunoprecipitated from these lysates were performed as previously described [55]. The phosphorylation substrate was recombinant GSK3α/β (Cell Signaling Technology, cat. no. 9237), and phosphorylation was detected with an antibody specific for GSK3β phosphorylated at Ser9. Western blots of the same lysates were probed with the anti-Akt3 or an anti-pan-Akt antibody (loading control). Quantification of the Western blot bands was performed using ImageJ software (for details see Fig. 2 legend).

For the kinase assays the ratio between pGSK3 and Myc-AKT3 or Myc-AKT1 has been calculated and the ratio volume for the WT forms was set as 1. For pAKTs at Ser or Thr, the ratio between pAKT and AKT3 or between pAKT and pan-AKT has been calculated. The calculated volume for the WT for was set as 1.

### Molecular modeling

The structural effects of the Q61H mutation were explored with modeling, starting from the crystal structure of the Akt1 PH domain in complex with Ins(1,3,4,5)-tetrakisphosphate [56]. Molecular modeling was performed using the program CHARMM and the generalized Born with molecular volume (GBMV) approach as previously described [57, 58].

### Schizophrenia GWAS study

We analyzed the publicly available dataset of the large-cohort schizophrenia GWAS study to determine whether common polymorphisms link AKT3 to schizophrenia [28, 29]. These available data [29] were retrieved through the Ricopili portal (https://data.broadinstitute.org/mpg/ricopili/), and the p-values of SNP association with the AKT3 locus were plotted.

### Adverse psychiatric events in clinical trials of PI3K/AKT/mTOR pathway inhibitors, including M2698

We performed a comprehensive literature review of the neuropsychiatric effects of other PAM pathway inhibitors and surveyed the AE data from randomized controlled trials of these drugs published in the U.S. National Library of Medicine clinical trials database (www.clinicaltrials.gov). We focused on psychiatric AEs and categorized them based on clinical presentation. Serious and non-serious AEs were reported as the proportion of patients affected. The AEs were reviewed by a psychiatrist (AV) with expertise in cancer clinical trials. The events were grouped into four general categories (impaired reality testing; delirium; mood disorders; other). We also systematically reviewed the database of patients with advanced cancer treated on a phase I study of the brain-penetrant p70S6K/AKT1/AKT3 inhibitor M2698 as monotherapy (*n* = 62) and in combination with tamoxifen (*n* = 26) or trastuzumab (*n* = 13) for all treatment-emergent AEs of the system organ class psychiatric disorders [34].

## Supplementary Information

**Additional file 1.** Supplemental Material.

## Data Availability

All the primary data are available.

## Abbreviations

AEs: Adverse events
CNS: Central nervous system
D2R: Dopamine 2 receptor
GBMV: Generalized Born with molecular volume
GSK3: Glycogen synthase kinase-3
GWAS: Genome-wide association studies
NRG1: Neuregulin-1
PAM: PI3K/AKT/mTOR
SNPs: Single nucleotide polymorphisms
