# Supplementary material for: AKT inhibition in the central nervous system induces signaling defects resulting in psychiatric symptomatology
Source: Cell Biosci. 2022 May 7;12:56. doi: 10.1186/s13578-022-00793-8 (PMC9080159; doi:10.1186/s13578-022-00793-8)
Supplement: Supplementary file 1 — Additional file 1. Supplemental Material. [file 13578_2022_793_MOESM1_ESM.docx]

**SUPPLEMENTAL MATERIAL**

**Supplemental Methods**

**Site-directed mutagenesis.** The purified DNA was used to transform *E.coli* DH5-alpha using standard procedures, and the mutation in the isolated clones was confirmed by sequencing. For the mutagenesis experiments we employed the following primers: For the mutagenesis experiments we employed the following primers: for AKT1 Q61H primer1: 5'-gctctgtcttcatcagatggcattgtgccactg-3' and primer2: 5'-cagtggcacaatgccatctgatgaagacagagc-3', and for AKT3 Q60H primer1: 5'-tggtcgttctgttttcattaaatgacattttgccactgagaag-3' and primer2: 5’-cagtggcacaatgccatctgatgaagacagagc-3’.

**Molecular modeling.** Molecular modeling was performed using the program CHARMM for an all-atom force field potential energy description of the protein-ligand complexes. The generalized Born with molecular volume (GBMV) approach was used for all energy evaluations to ensure physically rigorous evaluation of electrostatic potential energy components from solvent [1, 2]. The complex was initially energy-minimized in CHARMM using a GBMV implicit solvent model. The nonbonded interactions were calculated using an 18 Å cutoff such that the VDW, electrostatic, and GBMV solvation terms were switched off smoothly from 16 to 18 Å. Prior to the initialization of velocities, the complex was minimized for 100 steps using steepest descents for well-behaved temperature equilibration from random seeds. The system was slowly heated from 0 to 300 K over the course of 50 ps, and production dynamics were performed at 300 K for 100 ps. Langevin dynamics was used to control the temperature of the system with a friction coefficient (beta = 5.0 ps -1) for all non-hydrogen atoms. The bond lengths involving hydrogen atoms were fixed using the SHAKE algorithm. Velocities were rigorously integrated with a short 1.0 fs time step to minimize any integration error. Coordinates were saved every 100 steps for analysis.

Side chain rotamers of either Q61 or H61 were built using the backbone (ϕ,ψ)-specific Dunbrack rotamer library (2002 version) [3-5]. In building side chains, all of the possible non-clashing rotamers were considered, and polar atom distances between residue 61 and R76 were calculated. While placing the most favorable rotamers at this specific residue position, it was assumed that all other residues remained rigid. Retaining the crystallographic side chain conformation of R76, the most favorable Q61 side chain rotamer from the library, forms very favorable polar interactions with the carbonyl group of the Q61 side chain and the guanidino side chain of R76 (with a distance of 2.9 Å between Q61@OE1 and R76@NH1). All of the possible side chain conformations for residue 61 as Gln and His were enumerated. Four of the most common rotamers of Gln were able to form similar interactions, with distances ranging from 2.9 to 3.2 Å (between Q61@OE1 and R76@NH1). Alternatively, for His there were only six possible non-clashing rotamers. Only two of these, which were less frequent and more uncommon rotamers, were able to form close polar interactions with much larger minimum distances between equivalent polar atoms in the range of 4.1-4.2 Å (between H61@ND1 or H61@NE2 and R76@NH1).

Molecular modeling data from two independent and complementary approaches demonstrate that the carbonyl group of the Q61 side chain may form favorable polar interactions with the guanidino side chain of R76. Such side-chain interactions were identified in short molecular dynamics (MD) simulations exploring the dynamics of the PH domain in its native-state and subsequent conformational changes in the D3-phosphorylated phosphoinositide binding pocket. This observation, which was initially based on MD simulations, was also independently confirmed by analyzing the crystal structure using a side-chain rotamer library. Several of the most common Q61 side chain library rotamers, were also able to form similar favorable interactions with short interaction distances between polar atoms. However, histidine side chain rotamers from the library were not able to form equivalent favorable interactions. Based on the combination of these observations from analysis of MD simulations and rotamer libraries, we hypothesize that H61 may not be able to form the favorable side-chain interactions Q61 forms with R76. Analysis of MD simulation trajectories show how observed structural changes to the conformational state of the Q61H binding pocket residues K14 and R86, which directly participate in ligand binding, support the hypothesis that Q61H may reduce the binding affinities for D3 phosphorylated phosphoinositides.

**Supplemental Results**

## Psychiatric adverse events of selected patients treated with M2698

**Delusion**. One patient on treatment developed paranoid delusions attributed to the study drug. The patient was a 48-year-old man with malignant melanoma of the skin of the left neck and scalp. He was initially treated with wide local excision and sentinel lymph node dissection, followed by adjuvant radiation therapy to the scalp and neck (30 Gray, 5 fractions). A year later he developed biopsy-confirmed metastatic melanoma in the lung. He was treated by participating in multiple clinical trials to which his disease was refractory.

Three years after the initial diagnosis the patient was started on M2698 (320 mg daily orally, 1 cycle = 21 days) because molecular testing of tumor tissue collected at the time of the initial diagnosis had detected a *PIK3CA* E81K mutation. M2698 treatment was uneventful until day 11 of cycle 2, when the patient presented to the Emergency Center because of transient expressive aphasia, delusion, and indecision. He had difficulty understanding verbal communication, including his own speech; this gradually cleared. He was anxious and paranoid related to his work status and concerns that co-workers were conspiring against him. He said that he realized that these thoughts were irrational, but he could not control them.

His medical history included hyperlipidemia and hypertension. He had no history of prior psychiatric illness or drug or alcohol abuse. He was divorced with no children and he worked as an office operations manager. At the time of the treatment, he took only the study drug and allopurinol. He was awake; alert; oriented to person, place, and time; his speech was fluent without dysarthria; and his attention and concentration were intact.  He could follow simple and 2-step commands. Cranial nerves II through XII were also intact, and deep tendon reflexes, motor examination, and sensation were normal.

He was admitted to the inpatient service. Complete blood counts, chemistry, and electrolytes were within normal limits.  Urine and blood toxicology studies, computed tomography (CT) and magnetic resonance imaging (MRI) of the brain, spinal fluid chemistry and cytology, and electroencephalography were all normal. A psychiatrist diagnosed paranoid delusion, likely secondary to M2698, which was temporarily discontinued, and the patient was treated with risperidone and supportive psychotherapy. He was also diagnosed with adjustment disorder with anxiety and was treated with lorazepam. A few days from the start of the treatment his symptoms significantly improved, except for the anxiety, which was increased, but was treated with partial success with clonazepam.

Following the completion of cycle 2 the patient was asymptomatic and imaging studies demonstrated stable disease by RECIST 1.1 (Response Evaluation Criteria In Solid Tumors). Cycle 3 started with 200 mg daily (decreased from 320 mg). On cycle 3, day 3, he experienced symptoms of anxiety, panic, and tachycardia.  This time M2698 treatment at the reduced dose was not discontinued and it was given along with risperidone and lorazepam. Following this regimen, he was able to complete a total of 4 cycles of treatment without recurrence of delusion. Treatment was discontinued after the 4^th^ cycle because of disease progression. Following this, he enrolled in another clinical trial, without recurrence of delusion. Risperidone was gradually tapered to discontinuation, and the patient was treated with escitalopram and clonazepam for adjustment disorder. He died 8 months later with progressive disease.

**Nightmares.** Another patient treated at the 320 mg daily M2698 dose reported nightmares. The patient was a 57-year-old man with mucoepidermoid carcinoma of the right parotid salivary gland. He underwent radical parotidectomy followed by adjuvant radiation therapy (60 Gy, 30 fractions). One year later, he developed biopsy-confirmed lung metastases. He was initially treated with docetaxel and carboplatin and his disease progressed after 2 cycles. He was enrolled in the M2698 clinical trial because his tumor had a PIK3CA mutation (NM_006218.2(*PIK3CA*): c.328_330del p.E110del in exon 2). The patient tolerated treatment well. However, starting on day 3 of cycle 4, the patient developed nightmares, which he described as "frightening, hyper-real dreams.”  The dreams disappeared when M2698 was given at the reduced dose of 200 mg daily. Treatment was discontinued after 13 cycles because of disease progression (RECIST 1.1).

## Patients with preexisting psychiatric disorders may be treated with PI3K-AKT inhibitors with close psychiatric monitoring

Given the psychiatric complications observed in the above patients, a patient who met the criteria for the study but had a history of chronic bipolar disorder was enrolled with caution and under close psychiatric monitoring. The patient was a 46-year-old woman with metastatic moderately differentiated endometrioid carcinoma of the ovary, whose disease was refractory to standard treatment. Molecular profiling revealed that her tumor carried the PIK3CA L456R mutation. The patient was treated with 240 mg M2698 for a total of 6 cycles, with no psychiatric complications. Treatment was ultimately discontinued because of disease progression.

A 47-year-old female with a mammary adenocarcinoma carrying an amplified AKT3 gene was treated with 200 mg M2698 in combination with tamoxifen. She had documented pre-existing bipolar disorder for more than 20 years and was treated with mood stabilizing medication. There was no exacerbation of her psychiatric symptoms during treatment. M2698 was discontinued after 10 weeks due to disease progression.

**Supplemental Table 1. Antibodies used for immunoblotting**

| **Target** | **Cat. No.** |
| --- | --- |
| AKT1 | 2938 |
| AKT3 | 8018 |
| AKT (pan) | 4685 |
| p-AKT (Thr 308) | 4056 |
| p-AKT (Ser 473) | 4060 |
| p-PGSK-3α/β | 8566 |
| MYC tag | 2276 |
| TUBULIN | T5168 |
| GAPDH | MAB374 |

All antibodies were manufactured by Cell Signaling except for Tubulin and GAPDH that were manufactured by Sigma and Millipore, respectively.

## Supplemental Table 2: Patients reporting multiple (≥3) psychiatric AEs with M2698 treatment

| **Cohort** | **Dose of M2698, mg/day** | **Patient ID** | **Psychiatric AEs (n)** | **Psychiatric AEs preferred term  Grade NCICTCAE = (G)** | | | | **Medical history psychiatric events**  **(Grades provided for ongoing events)** |
| --- | --- | --- | --- | --- | --- | --- | --- | --- |
| Monotherapy | 320 | 15 | 5 | Anxiety (G2) | Abnormal dreams (G1)  Insomnia (G2) | Depression (G2) | Delusion (D2) | None |
| Tamoxifen | 80 | 17 | 4 | Anxiety (G1) | Paranoia (G1) | Reading disorder (G1) | Anxiety (G1) | Anxiety  Depression  Insomnia (G1) |
| Trastuzumab | 160 | 19 | 4 | Anxiety (G2)  Emotional distress (G1) | Insomnia (G1) | Depression (G1) |  | Anxiety (G1)  Insomnia |
| Trastuzumab | 160 | 26 | 3 | Anxiety (G1) | Insomnia (G2) | Depression (G2) |  | none |
| Trastuzumab | 160 | 31 | 5 | Euphoric mood (G2)  Mania (G3) | Abnormal dreams (G1) | Hallucination (G1) | Paranoia (G1) | Depression (G1) |

**Supplemental Table 3. Selected PAM pathway inhibitors tested in clinical trials**

| **Inhibitor type** | | | **Agent** | **FDA Approval** |
| --- | --- | --- | --- | --- |
| **Pan-PI3K Inhibitors:**  **Inhibit all the 4 class I PI3K catalytic subunits: p110α, p110β, p110γ, p110δ** | | | Buparlisib (BKM120) |  |
|  |  |  | Pilaralisib (XL147) |  |
|  |  |  | Pictilisib (GDC-0941) |  |
|  |  |  | Dactolisib (BEZ235) |  |
| **Isoform-speciﬁc PI3K Inhibitors:**  **Selectively inhibit catalytic subunits of class I PI3K** | **PI3Kα** | | Alpelisib (BYL719) | 2019, HR+ MBC |
|  |  |  | Taselisib (GDC-0032) |  |
|  |  |  | Serabelisib (PETRA Pharma) |  |
|  | **PI3Kβ** | | GSK2636771 (GSK) |  |
|  | **PI3Kδ** | | Umbralisib (TG Therapeutics) | NDA accepted 2020 for MZL |
|  |  |  | Idelalisib (GILEAD) | 2014, NHL |
|  |  |  | AMG-319 (AMGEN) |  |
|  |  |  | Parsaclisib (INCYTE) |  |
|  | **PI3Kδ/α** | | Copanlisib (BAY 80-6946) | 2017, FL |
|  | **PI3Kδ/γ** | | Tenalisib (Rizen Pharm.) |  |
|  |  |  | Duvelisib (VERASTEM) | 2018, CLL/SLL, FL |
|  | **PI3Kβ/δ** | | KA2237 (KARUS Therapeutics) |  |
| **Pan-Akt Inhibitors:**  **Inhibit all Akt isoforms (Akt 1, 2, 3)** | | | MK-2206 |  |
|  |  |  | Uprosertib (GSK2141795) |  |
|  |  |  | Ipatasertib (GDC-0068) |  |
|  |  |  | Capivasertib (AZ) |  |
|  |  |  | AZD5363 |  |
|  |  |  | BAY1125976 |  |
| **mTOR Inhibitors** | |  | Sapanisertib (TAKEDA) |  |
|  |  |  | Everolimus | 2009, Kidney  2012, Pancreatic NET  2012, HR+ BC |
|  |  |  | Temsirolimus | 2007, RCC, Kidney |
| **Dual Inhibitors** | | **p70S6K/AKT1/3** | M2698 |  |
|  |  | **mTOR/PI3K** | Gedatolisib |  |
|  |  |  | Dactolisib |  |
|  |  |  | Paxalisib |  |
|  |  |  | Bimiralisib |  |

*Abbreviations*: CLL, chronic lymphocytic leukemia; FL, follicular lymphoma; HR+ BC, hormone-receptor positive breast cancer; MBC, metastatic breast cancer; MZL, marginal zone lymphoma; NDA, new drug application; NET neuroendocrine tumor; NHL, non-Hodgkin’s lymphoma; SLL, small lymphocytic lymphoma; RCC, renal cell carcinoma.

**REFERENCES**

1. Lee MS, Feig M, Salsbury FR, Jr., et al. New analytic approximation to the standard molecular volume definition and its application to generalized Born calculations. J Comput Chem. 2003;24(11):1348-56.

2. Feig M, Onufriev A, Lee MS, et al. Performance comparison of generalized born and Poisson methods in the calculation of electrostatic solvation energies for protein structures. J Comput Chem. 2004;25(2):265-84.

3. Bower MJ, Cohen FE, Dunbrack RL, Jr. Prediction of protein side-chain rotamers from a backbone-dependent rotamer library: a new homology modeling tool. J Mol Biol. 1997;267(5):1268-82.

4. Dunbrack RL, Jr. Rotamer libraries in the 21st century. Curr Opin Struct Biol. 2002;12(4):431-40.

5. Dunbrack RL, Jr., Cohen FE. Bayesian statistical analysis of protein side-chain rotamer preferences. Protein Sci. 1997;6(8):1661-81.
